# Supplementary material for: The pattern of lung function tests in children with sickle cell disease: A case-control study
Source: PLoS One. 2025 Jul 28;20(7):e0329064. doi: 10.1371/journal.pone.0329064 (PMC12303343; doi:10.1371/journal.pone.0329064)
Supplement: Attachment — Submitted filename: FeNO in SCD kids review.docx [file pone.0329064.s001.docx]

This case-control study investigates pulmonary function and fractional exhaled nitric oxide (FeNO) levels in children and adolescents (aged 6–20 years) with stable sickle cell disease (SCD) compared to healthy controls. The study aims to understand the evolving pattern of lung function in children and adolescents with SCD and explore the potential role of FeNO as a biomarker for airway inflammation and a predictor of acute chest syndrome (ACS) risk. The measurement of FeNO adds a novel dimension to the study, linking inflammation (as reflected by FeNO) to pulmonary function in SCD.

**Introduction:**

75-78: Nitric oxide (NO) is produced by red blood cells (RBCs) and is crucial for the process of

vasodilation. In sickle cell disease (SCD), the functionality of RBCs is compromised, which

adversely affects NO activity. This impairment contributes to the exacerbation of symptoms

during vaso-occlusive crises, creating a detrimental cycle. [8]

The authors may do well to highlight the different cellular sources of NO and the primary contributor to the NO measured as FeNO.

**Methods:**

FeNO Measurement:

While FeNO is a valuable biomarker for airway inflammation, the methodology does not explicitly describe how FeNO levels were measured or standardized. Clarifying the protocol (e.g., device used, number of measurements, exhalation flow rate) would enhance reproducibility.

Lack of Blinding:

The study does not mention whether researchers conducting PFTs or analyzing data were blinded to the case/control status. Lack of blinding could introduce observer bias.

Adjust for Multiple Comparisons:

Use corrections for multiple comparisons (e.g., Bonferroni) to reduce the risk of Type I errors.

**Results:**

The study reports a weak positive correlation between hydroxyurea duration and disease severity, but this could reflect confounding by indication (i.e., more severe cases are more likely to receive hydroxyurea).

Instead of multiple correlation analysis, the authors could consider a multivariate analysis like regression to control for other variables.

**Discussions:**

FeNO as a Biomarker:

While FeNO is a useful biomarker for Th2-mediated eosinophilic airway inflammation, its role in SCD-related lung disease is not well-defined. The study assumes FeNO reflects general inflammation, which may not be accurate. Additional biomarkers (e.g., sputum eosinophils, CRP, IL-6) could provide a more comprehensive assessment of airway inflammation.

The authors initially suspected that FeNO levels would be higher in SCD patients compared to controls, due to their known respiratory issues, general inflammation, and that FeNO could potentially be use as a diagnostic and treatment monitoring tool like in eosinophilic asthma. However, their findings did not support this. Authors continue to highlight that this may be because of NO depletion in SCD. However, The primary source of elevated NO in asthma is iNOS, which is upregulated in airway epithelial cells and inflammatory cells (e.g., eosinophils, macrophages) in response to inflammation. Authors should discuss potential reasons for conflicting FeNO results across multiple studies, such as differences in patient populations, disease states, or measurement techniques.

Lines 254-256: Thirdly, our analysis revealed that a younger

onset of the disease is associated with increased severity and a mixed or restrictive pattern

rather than an obstructive one, a finding that has not been previously reported.

It’s unclear what younger onset of disease means as all patients had SCD at birth.

Lastly, we examined the correlation between elevated FeNO levels and the frequency of blood

transfusions, thereby elucidating the underlying pathophysiological mechanisms at the level

of red blood cells.

The authors are not clear on how blood transfusion history was obtained. It would be nice to control for blood transfusions or give FeNO values for transfused vs transfused cases.

Tables/Figures need description

Figure 2 needs a legend
