# Supplementary material for: The pattern of lung function tests in children with sickle cell disease: A case-control study
Source: PLoS One. 2025 Jul 28;20(7):e0329064. doi: 10.1371/journal.pone.0329064 (PMC12303343; doi:10.1371/journal.pone.0329064)
Supplement: Attachment — Submitted filename: Response to Comments.docx [file pone.0329064.s004.docx]

Dear reviewers

We are grateful for your time and effort in reviewing out manuscript. Hereby we attach the response to your comments.

| S.no. | Comment | Response |
| --- | --- | --- |
| 1 | Please ensure that your manuscript meets PLOS ONE's style requirements, including those for file naming. The PLOS ONE style templates can be found at <https://journals.plos.org/plosone/s/file?id=wjVg/PLOSOne_formatting_sample_main_body.pdf> and <https://journals.plos.org/plosone/s/file?id=ba62/PLOSOne_formatting_sample_title_authors_affiliations.pdf> | Made necessary changes in accordance to PLOS One guidelines |
| 2 | Your ethics statement should only appear in the Methods section of your manuscript. If your ethics statement is written in any section besides the Methods, please move it to the Methods section and delete it from any other section. Please ensure that your ethics statement is included in your manuscript, as the ethics statement entered into the online submission form will not be published alongside your manuscript | Made the necessary changes according to your guidance |
| 3 | We note that you have indicated that there are restrictions to data sharing for this study. For studies involving human research participant data or other sensitive data, we encourage authors to share de-identified or anonymized data. However, when data cannot be publicly shared for ethical reasons, we allow authors to make their data sets available upon request. For information on unacceptable data access restrictions, please see <http://journals.plos.org/plosone/s/data-availability#loc-unacceptable-data-access-restrictions> | We have uploaded the anonymised data |
| 4 | The authors may do well to highlight the different cellular sources of NO and the primary contributor to the NO measured as FeNO | We have changed the introduction majorly to accommodate the requirements |
| 5 | While FeNO is a valuable biomarker for airway inflammation, the methodology does not explicitly describe how FeNO levels were measured or standardized. Clarifying the protocol (e.g., device used, number of measurements, exhalation flow rate) would enhance reproducibility | Clarification to the protocol added in the methods |
| 6 | The study does not mention whether researchers conducting PFTs or analyzing data were blinded to the case/control status. Lack of blinding could introduce observer bias. | Required changes made in the methodology |
| 7 | Instead of multiple correlation analysis, the authors could consider a multivariate analysis like regression to control for other variables. | Analysis done and added to the results |
| 8 | Authors should discuss potential reasons for conflicting FeNO results across multiple studies, such as differences in patient populations, disease states, or measurement techniques. | We have deeply examined this issue and mentioned in the revised discussion |
| 9 | It’s unclear what younger onset of disease means as all patients had SCD at birth. | We identify the problem in the statement and we have corrected ourselves |
| 10 | The authors are not clear on how blood transfusion history was obtained. It would be nice to control for blood transfusions or give FeNO values for transfused vs transfused cases. | We have corrected this problem in the methods section |
| 11 | Restrictive lung disease is only able to be diagnosed by obtaining lung volumes, which was not done here. A restrictive pattern can be suggested by spirometry but to truly diagnose restriction it needs to be confirmed with lung volumes, and often a “restrictive pattern” does not end up leading to a true restriction diagnosis when you obtain lung volumes. | We identified the error made by us. We have therefore replaced restrictive disease with pattern in all of the manuscript wherever necessary |
| 12 | I would recommend changing the title of the paper and keywords because we aren’t talking about diagnosed restrictive lung disease and that is what is implied. I would also change anywhere in the paper that implies a restrictive lung disease or mixed defect diagnosis instead of a pattern. This should also be mentioned as a limitation in the discussion. | Point well taken and title changed |
| 13 | I would recommend adding if all tests met acceptability and reproducibility prior to being included in this study/analysis. | Amendment made in the methods section |
| 14 | I am confused on what you are using to define obstruction. In the second paragraph of your methodology (lines 114-116), you say an obstructive defect is characterized by a reduced FEV1/FVC ratio of <70% or below the 5th percentile for adolescents (in the paper you cite [11] this says “adults” not “adolescents” so I would make sure to correct that, along with the restrictive pattern FVC <5th percentile (lines 117-118) in “adults” not “adolescents”), and <85% for ages 5-18 years. It is not clear to me which of these numbers you used (<70%, <5th percentile, or <85%) if you could clarify, especially since the age range you chose is 6-20 yo so includes patients in both age groups <18 years old and >18 years old. | Made the changes in the methods and have clarified the language |
| 15 | Specify in the 1st paragraph of the methodology (lines 98-99) how long the patients were in a stable state of health without pain, crises, or acute illnesses – was it one week, one month, one year? | Corrections made |
| 16 | 3. In the 7th paragraph of the results (lines 161-163) there is interpretation of the results: “this probably hints…” but interpretation of the results should be reserved for the discussion, with the results section simply detailing the results of your analysis.  4. In the 2nd paragraph of your discussion, you say 74% of patients from Hulke [13] exhibited a restrictive pattern, but in reviewing the paper that number is 24% (line 186). I am also not sure how your findings stand in contrast, especially since the studies are in different age groups, so I would highlight that and discuss more.  5. I am having trouble connecting most of the 3rd paragraph of your discussion (lines 198-214) back to the paper – including the sentence on LDH levels. I am not entirely sure why you chose the include studies that you did, and how they relate to what your study looks at. Would recommend connecting this paragraph and the studies that you mention back to relevance and findings from your study.  6. In the 7th paragraph of the discussion, I’m confused by the sentence, “Secondly, we successfully utilized fractional exhaled nitric oxide (FeNO) as a proxy for obstructive dysfunction, which corroborated our findings, as we observed a predominance of restrictive patterns over obstructive ones” (lines 252-254). I don’t know where that comes from in your results to use FeNO as a proxy for obstruction, and I’m not sure how that would relate to a predominance of restrictive patterns. Please clarify.  7. Try to organize the discussion in a more logical way. For example, put all limitations together. You mention this was a non-funded study with limited time and possible sampling bias in the first paragraph, but more of the limitations are listed later in the discussion. Also, try to incorporate your study results with the papers that you discuss as mentioned above to connect your study more with current literature.  8. Another limitation you should mention in your discussion is the exclusion of children who were being treated with inhaled bronchodilators, since this group may have represented even more severe pulmonary disease.  9. Since you don’t talk about incentive spirometry at all in the paper I wouldn’t bring it in for the last sentence of your discussion (lines 265-266) since you haven’t established how that would related to your findings. It might be better to say you would want to connect the parameters that you studied with respiratory symptoms and other clinical markers of disease, or something else.  10. I would read through again and make sure to edit for grammar since there are quite a few errors throughout. For example, in the last paragraph of the results the sentence, “more the number of blood transfusions, lesser were the FeNO levels” does not make sense as it is written. You also need to make sure that all abbreviations are written out originally, and then you don’t need to re-define them later. For example, in the introduction PFTs, ACS, and Th2 are not defined before they are used as abbreviations, but FeNO is defined twice and SCD defined three times. In the 3rd paragraph of the methodology, you repeat FEV1 in “FEV1 percent of predicted FEV1” that only needs to say FEV1 once.  11. Citations should be moved to the end of the sentence(s) that they connect with, not the beginning of the sentence. | We have revised the entire discussion as the previous one was deeply flawed. |
