# Supplementary material for: The pattern of lung function tests in children with sickle cell disease: A case-control study
Source: PLoS One. 2025 Jul 28;20(7):e0329064. doi: 10.1371/journal.pone.0329064 (PMC12303343; doi:10.1371/journal.pone.0329064)
Supplement: Attachment — Submitted filename: PFT abnomralities in Indian SCD children.docx [file pone.0329064.s003.docx]

**Abstract**

**Background**

- "definitive change" is vague. Suggest using "widespread involvement."
- Consider linking inflammation and lung dysfunction more explicitly.

**Methods**

- Define devices more clearly; “hand-held device” is vague.
- “Data was collected and analysed.” → “Data were collected and analyzed.”

**Results**

- CI for FEV1/FVC appears to have an error: duplicate "95% CI."
- Better organization would improve readability: group findings (e.g., PFT differences, clinical correlations).

Example:

**Results**
Children with SCD had significantly lower pulmonary function values compared to controls:

- FEV1 median difference: 33.5% (95% CI: 27.2–38.0; p<0.0001)
- FVC: 25.4% (95% CI: 30.0–32.25; p<0.0001)
- FEV1/FVC: 0.088 (95% CI: 0.075–0.083; p<0.0001)
- PEFR: 24.8% (95% CI: 16.38–33.8; p<0.0001)
- FeNO: 8.17 ppb (95% CI: 5.77–12.65; p<0.0001)

Pulmonary function abnormalities were associated with younger age (p=0.0022). Age (p=0.0075) and weight (p=0.0093) were significantly associated with PFT severity, while blood transfusion frequency, hydroxyurea duration, and FeNO levels were not.

**Conclusions**

- "FeNO levels does not..." → should be "do not."
- Clarify "severity of illness" – based on PFT pattern?

Introduction:

• **Clarity & Grammar**:

- "individuals SCD" should be "individuals with SCD."
- The phrase "mainly found in the lower parts of the lungs" is informal—could be more technical.

• Better explain study aim

**Results:**

Table 5 is missing.

Table 6 implies that the dependent variable was a continuous variable and not the categorical severity pattern shown in Table 4.. The authors do not clarify what this measure of severity was, FEV1?

interpretation of the analysis.

Take for instance, Age, on face value, one would interpret it as follows:

Coefficient: -0.0658

- **Interpretation**: Older age is associated with **worsening** of the dependent variable (lower PFT scores), and this is **statistically significant** (*p = 0.00749*).
- **Clinical Insight**: Lung impairment may worsen with age in SCD

However, the authors in the discussion now state: "It was noted that older age was significantly linked to either a normal or obstructive pattern. In the multivariate analysis, weight and age exhibited a negative correlation with the severity of illness."

Its important to emphasize the results of the multivariate analysis and downplay the findings from simple comparisons, especially when they conflict with the multivariate analysis.

The phrase *"risk difference in our cohort of patient is 0.83"* needs clarification—what risk is being referenced?

Tables and figures are labeled, but data is hard to follow due to repeated or inconsistent labels.

Mentioning the Bonferroni correction is excellent, but more context is needed: how many comparisons were made to justify the adjusted α?

Discussion:

• Strengthen **the connection between multivariate regression and the discussion**

While your regression analysis showed significant effects of age and weight, the discussion does not fully integrate these findings with the broader conclusions. A more explicit link between the statistical results and disease severity would improve coherence.

•There is too much discussion on unrelated studies. …Kassim …can be removed

Lines 230: We however did not do definitive diagnosis for restriction with diffusion studies, so the findings of our study are mainly ‘pattern’-based.

**Lung restriction** is diagnosed primarily by **lung volume measurements**. DLCO helps characterize restrictive patterns but cannot diagnose restriction on its own.

•

Discussion can be more concise.
