# Supplementary material for: The pattern of lung function tests in children with sickle cell disease: A case-control study
Source: PLoS One. 2025 Jul 28;20(7):e0329064. doi: 10.1371/journal.pone.0329064 (PMC12303343; doi:10.1371/journal.pone.0329064)
Supplement: Attachment — Submitted filename: PFT_abnomralities_in_Indian_SCD_children_auresp_2.docx [file pone.0329064.s005.docx]

| S. No. | Comment | Reply |
| --- | --- | --- |
| 1 | - "definitive change" is vague. Suggest using "widespread involvement." | Thank you for wording it correctly; the change has been made in the unmarked copy at 64 |
| 2 | - Consider linking inflammation and lung dysfunction more explicitly | Thank you for your observation, however due to limitation of words, we are unable to do so in the abstract, we however go into the details in the main article |
| 3 | - Define devices more clearly; “hand-held device” is vague. | In the unmarked copy line 72, we mention the name of the device. |
| 4 | - “Data was collected and analysed.” → “Data were collected and analyzed.” | Made the necessary change at line 72. Thank you for pointing out the obvious mistake. |
| 5 | - CI for FEV1/FVC appears to have an error: duplicate "95% CI." - Better organization would improve readability: group findings (e.g., PFT differences, clinical correlations).   Example:  **Results** Children with SCD had significantly lower pulmonary function values compared to controls:   - FEV1 median difference: 33.5% (95% CI: 27.2–38.0; p<0.0001) - FVC: 25.4% (95% CI: 30.0–32.25; p<0.0001) - FEV1/FVC: 0.088 (95% CI: 0.075–0.083; p<0.0001) - PEFR: 24.8% (95% CI: 16.38–33.8; p<0.0001) - FeNO: 8.17 ppb (95% CI: 5.77–12.65; p<0.0001)   Pulmonary function abnormalities were associated with younger age (p=0.0022). Age (p=0.0075) and weight (p=0.0093) were significantly associated with PFT severity, while blood transfusion frequency, hydroxyurea duration, and FeNO levels were not. | Thank you for suggesting a more robust method of representing out results. We have done the same in the abstract from line 73 to 83 |
| 6 | - "FeNO levels does not..." → should be "do not." | Made the change at line 85 |
| 7 | - "individuals SCD" should be "individuals with SCD." | Made the change at line 94 |
| 8 | - The phrase "mainly found in the lower parts of the lungs" is informal—could be more technical | Changed at line 92-93 |
| 9 | - Better explain study aim | “This study examines the PFT and FeNO results of children with SCD (ages 6 to 20 years) in comparison to age-, sex-, and height-matched individuals without SCD.”   - Lines 114 to 115 |
| 10 | Table 5 is missing. | We apologize for the oversight. We have added the table |
| 11 | Table 6 implies that the dependent variable was a continuous variable and not the categorical severity pattern shown in Table 4.. The authors do not clarify what this measure of severity was, FEV1? | The initial analysis for severity was based on the FEV1% of the expected. On reviewing literature we realized that severity is based on the z-scores of FEV1, therefore we have made the change and updated the analysis |
| 12 | interpretation of the analysis.  Take for instance, Age, on face value, one would interpret it as follows:  Coefficient: -0.0658   - **Interpretation**: Older age is associated with **worsening** of the dependent variable (lower PFT scores), and this is **statistically significant** (*p = 0.00749*). - **Clinical Insight**: Lung impairment may worsen with age in SCD   However, the authors in the discussion now state: "It was noted that older age was significantly linked to either a normal or obstructive pattern. In the multivariate analysis, weight and age exhibited a negative correlation with the severity of illness.” | Thank you for simplifying the method of description for us. We have made the necessary changes |
| 13 | Its important to emphasize the results of the multivariate analysis and downplay the findings from simple comparisons, especially when they conflict with the multivariate analysis. | We identify this issue and we have made the necessary amendment |
| 14 | The phrase *"risk difference in our cohort of patient is 0.83"* needs clarification—what risk is being referenced? | We identified that an odd’s ratio must be calculated for a case-control study and we have made the change accordingly |
| 15 | Tables and figures are labeled, but data is hard to follow due to repeated or inconsistent labels. | We have tried to resolve this issue to the best of our abilities |
| 16 | Mentioning the Bonferroni correction is excellent, but more context is needed: how many comparisons were made to justify the adjusted α? | 6 comparisons were made. Added in the text |
| 17 | • Strengthen **the connection between multivariate regression and the discussion**  While your regression analysis showed significant effects of age and weight, the discussion does not fully integrate these findings with the broader conclusions. A more explicit link between the statistical results and disease severity would improve coherence.  • Discussion can be more concise. | We have tried to do so in the new discussion |
| 18 | •There is too much discussion on unrelated studies. …Kassim …can be removed | Omitted as advised |
| 19 | Lines 230: We however did not do definitive diagnosis for restriction with diffusion studies, so the findings of our study are mainly ‘pattern’-based.  **Lung restriction** is diagnosed primarily by **lung volume measurements**. DLCOhelps characterize restrictive patterns but cannot diagnose restriction on its own. | Thank you for pointing out our oversight. We have made the necessary changes. |
