# Supplementary material for: The pattern of lung function tests in children with sickle cell disease: A case-control study
Source: PLoS One. 2025 Jul 28;20(7):e0329064. doi: 10.1371/journal.pone.0329064 (PMC12303343; doi:10.1371/journal.pone.0329064)
Supplement: Attachment — Submitted filename: Response to review.docx [file pone.0329064.s006.docx]

| Remark | Response |
| --- | --- |
| 1. Abbreviations should be written out when they are first encountered in the abstract and again in the paper. After that, those same abbreviations do not need to be re-defined later. For example, line 65 “PEFR” and line 84 “Th2” are not previously defined anywhere and should be written out as “peak expiratory flow rate”, and “T helper 2”, respectively. As another example, lines 81-82 “pulmonary function tests” should have “(PFTs)” after it but then not later in the paper, such as in line 171, 197, and 227, and the abbreviation “PFT” should be used like in line 206. This also occurs with defining “sickle cell disease (SCD)”, “lactate dehydrogenase (LDH)”, “hemoglobin (Hb)”, “nitric oxide (NO)”, and others. | We have reviewed the entire document and we have tried to stick to this format. We are indebted to you for pointing out these minute errors |
| 2. Use appropriate upper and lower case. For example, line 75 “Sickle Cell Disease”, and lines 77-78 “Vaso-Occlusive Crises” and “Acute Chest Syndrome”, should all be lower case. | We have made these changes |
| 3. Citations should be moved inside of the period. For example, line 77 should be “worldwide [1].” | We have made the changes throughout the document |
| 4. Throughout the paper there are a lot of places where there should be a space after a period before the start of the next sentence. Specifically, line 190 there needs to be a space between “in” and “dependent” because otherwise it reads “independent”, which is a different type of variable. It also should be “in the dependent” variable. | Made the necessary changes |
| 5. Lines 137-138 are awkwardly phrased. You could change it to, “Information on the history of blood transfusions and the duration of hydroxyurea administration was gathered from medication records for the cases.” | Made the changes |
| 6. Line 151 write out “eight” instead of “8”. | Made the changes |
| 7. Lines 191 and 204 FEV1 z-scores needs a dash, and “better” should be changed to something like “higher” since you are not discussing better or worse here but instead if they are higher or lower. Line 204 also should be “for longer” instead of “for more”. | Made the changes |
| 8. Line 223 should be “Research conducted…” or “A research study conducted…” instead of “A research conducted”. | Made the changes |
| 9. Line 274 “same holds true for FeNO” is not complete - recommend changing the sentence before to “PFTs and FeNO” or add “The” before “same”. | Made the changes |
| 10. Line 274 “it would not be unfair to assume” instead of “it will not be unjust to assume”. | Made the changes |
| 11. In the abstract lines 56-57 “acute or chronic cardiorespiratory conditions” should also be listed as an exclusion since you list that in lines 114-115. | Made the change at line 57 |
| 2. In line 77 specify “acute conditions that occur in patients with SCD” | Added the conditions as ACS, VOC, aplastic and hemolytic crises |
| 3. I am not able to find the information you discuss in lines 78-82 in the papers that you cite [4,5]. The definition of sickle cell chronic lung disease has evolved significantly since the Powars paper was published in 1988 and there is no one true entity “sickle cell chronic lung disease”. You could look at a more recent review such as “Updates in Pediatric Sickle Cell Lung Disease” by Gillespie published 2024 in Clin Chest Med. Additionally, in line 81, it is unclear if you mean 90% of children with SCD or of children with sickle cell chronic lung disease. | [5, now 4] Consistent with the results of pulmonary function testing, HRCT scans revealed mild basilar focal fibrosis, with fibrosis scores trending to increase with increased pulmonary artery systolic pressures ([Figures 3A and 3B](https://www.atsjournals.org/doi/full/10.1164/rccm.200610-1498OC)).  We have updated the Powars to Gillespie and made the corresponding changes in the introduction at lines 86-89 (marked copy) |
| 4. I am unsure why you are mentioning the sentence starting with “Consequently, markers…” lines 91-93 since you do not go on to discuss arginine in the paper again. Would recommend removing this. | Duly noted and made the necessary change |
| 5. In lines 118-119 I would recommend not mentioning specific limitations until the discussion and would move this down to the paragraph starting line 272. Instead, for lines 118-119, you could write something like, “The sample size was determined based on recruitment ability within the timeframe of the study”. | Duly noted and changes made |
| 6. Line 130 would not use “diagnosed with” but instead “characterized as having” | Duly noted and changes made |
| 7. Line 150 recommend “were excluded”, instead of “had to be excluded”. | Duly noted and changes made |
| 8. Line 159 recommend “group with patients diagnosed with SCD”, instead of “sickle cell group”. | Duly noted and changes made |
| 9. Line 182 “The heat chart…” should be deleted since that sentence is copied twice in a row. | Duly noted and changes made |
| 10. Line 212 specify “abnormal findings in this study” if that is what you are referring to. | Mentioned the obstructive, restrictive or mixed patterns on PFT taken as abnormal |
| 11. Lines 221 and 286 these are “lung volume” studies that would diagnose restrictive lung disease, not “diffusion” studies | We have changed the item to lung-volume study for definitive diagnosis |
| 12. Line 224 need to clarify “predominant abnormality” in who? Patients with SCD? | We have added the clarification |
| 13. Line 227 instead of “them” should say “this study by Arteta”. | Duly noted and changes made |
| 14 Lines 228-229 I’m not sure that we can declare that this finding supports Arteta’s hypothesis that hemolysis may be linked to the obstructive pattern just because it is occurring in older patients | Duly noted and changes made |
| 15. Line 237 clarify if you are talking about your own study again with the sentence “additionally…”. I am also unsure how this does not align with Adekile’s hypothesis in the following sentence. I re-read this paragraph a few times and I am still unsure of what is intended. Recommend re-writing these few sentences to be clearer. | A similar study was done by Adekile et al on Kuwaiti children [20]. The research indicated that the correlation coefficients between FEV1 and various anthropometric measurements demonstrated a significant relationship with height (r = 0.9, p < 0.001) and weight (r = 0.8, p < 0.001). Our findings were consistent, showing that weight was notably linked to improved FEV1 z-scores. In our study children who were treated with hydroxyurea for an extended duration exhibited enhanced FEV1 z-scores. Hydroxyurea is expected to increase fetal Hb in SCD, likely reducing hemolysis [21]. This does not align with the findings by of the study Adekile et al [20] as they did not demonstrate any significant correlation with Hb, fetal Hb, reticulocytes, total bilirubin, or LDH. We, as stated earlier, strive to study the hemolysis in more details in forthcoming studies. |
| 16. Line 269 confusing what you mean by “earlier onset of the disease” since SCD is a genetic condition, so all patients have it at birth. Please clarify. | earlier onset of the pulmonary component of the disease |
| Another limitation that should be included in the discussion paragraph starting line 272 is excluding children using inhaled bronchodilators and those with any acute or chronic cardiorespiratory conditions identified. Those are important patients to potentially consider as they may represent those with more severe pulmonary disease and would possibly change the outcome of the study. | Our study had several limitations as well. We had excluded children using inhaled bronchodilators and those with any acute or chronic cardiorespiratory conditions. Those are important patients to potentially consider as they may represent those with more severe pulmonary disease and would possibly change the outcome of the study. |
| Would recommend putting all future directions in the same paragraph below in 280-282 taken from lines 229-231 and 240-241. | Added all future direction at the end as recommended |

1. We have submitted our raw data as supplementary document for your kind perusal
2. an expert in sickle cell-related lung disease has requested additional modifications, such as clarifying how restrictive and obstructive lung patterns were determined and to avoid using that terminology if the distinction is not clear. : We mention it in the methodology section as to how we came to the conclusion of the obstructive and restrictive patterns.
